# Rare Presentation of Necrotic Appendicitis and Small Bowel Obstruction in an Elderly Man: De Garengeot Hernia

**DOI:** 10.7759/cureus.106601

**Published:** 2026-04-07

**Authors:** Luis Felipe Mondardo Spengler, Gustavo Tamura, William Gabriel Bohrer, Germano Andrade Maranduba

**Affiliations:** 1 Department of Surgery, Hospital Governador Celso Ramos, Florianópolis, BRA

**Keywords:** acute appendicitis, case report, de garengeot hernia, femoral hernia, general surgery

## Abstract

De Garengeot hernia, characterized by the presence of the vermiform appendix within the femoral hernia sac, is a rare condition often diagnosed only intraoperatively. The incidence of acute appendicitis within a De Garengeot hernia is even more uncommon, and its clinical presentation may be atypical, making preoperative diagnosis difficult. This report describes the case of a 67-year-old male patient with multiple comorbidities, including an abdominal aortic aneurysm, who presented with right inguinal pain and bulging, culminating in the intraoperative diagnosis of a De Garengeot hernia with acute appendicitis and tip necrosis. The surgical approach involved appendectomy and hernia repair without mesh due to the risk of infection. The patient evolved favorably, highlighting the importance of high clinical suspicion and appropriate surgical management for this rare and potentially serious condition.

## Introduction

De Garengeot hernia is a rare clinical entity defined by the presence of the vermiform appendix within a femoral hernia sac. First described by René Jacques Croissant de Garengeot in 1731, its incidence is estimated at 0.5% to 5% of all femoral hernias and only 0.08% to 0.13% of all abdominal hernias [1]. Acute appendicitis within a De Garengeot hernia is even more uncommon, occurring in approximately 0.08% to 0.13% of femoral hernia cases, and its clinical presentation can be challenging, mimicking other conditions such as lymphadenitis, soft tissue abscess, or simple incarcerated hernia [2]. Preoperative diagnosis is infrequent, with most cases identified during surgical exploration.

Surgical management of De Garengeot hernia with acute appendicitis requires special considerations, particularly regarding appendectomy and the type of hernia repair. The presence of inflammation or infection in the operative field generally contraindicates the use of prosthetic mesh, favoring anatomic repairs to minimize the risk of infectious complications [1]. This case report aims to describe a case of De Garengeot hernia with acute appendicitis and tip necrosis in an elderly patient with multiple comorbidities, discussing the diagnostic challenges and particularities of surgical management in accordance with current literature.

## Case presentation

Patient information and clinical presentation

A 67-year-old male patient was admitted to Hospital Governador Celso Ramos (Santa Catarina, Brazil) on October 23, 2025, with complaints of right inguinal pain and bulging for one day. He reported pain in the region for approximately one week, with significant worsening over the past 24 hours. Associated with these symptoms, he presented with three episodes of vomiting and constipation for four days, denying fever. The patient had a history of intellectual disability, chronic obstructive pulmonary disease (COPD), and an infrarenal abdominal aortic aneurysm measuring 6.4 × 6.2 cm, recently diagnosed. His continuous medications included carbamazepine, acetylsalicylic acid (ASA), amitriptyline, rosuvastatin, and alenia. He had previously undergone left inguinal hernioplasty and umbilical hernioplasty. He was a smoker.

On physical examination, the abdomen was somewhat distended and tender to deep palpation. A supposedly right inguinal incarcerated hernia was identified, tender to palpation.

Diagnostic investigation

Laboratory tests performed on 10/23/2025 are shown in Table 1.

**Table 1:** Laboratory results

Laboratory Parameter	Patient Value	Reference Range	Status
Hemoglobin	15.5 g/dL	13.5-17.5 g/dL	Normal
Hematocrit	46%	41-53%	Normal
White Blood Count	19,370/µL	4,500-11,000/µL	Elevated
Leukocyte Differential	Left shift present	Normal distribution	Abnormal
Platelets	282,100/µL	150,000-400,000/µL	Normal
Urea	74 mg/dL	7-20 mg/dL	Elevated
Creatinine	1.58 mg/dL	0.7-1.3 mg/dL	Elevated
Sodium	138 mEq/L	136-145 mEq/L	Normal
Potassium	4.6 mEq/L	3.5-5.0 mEq/L	Normal
C-Reactive Protein	153 mg/L	<10 mg/L	Elevated
D-Dimer	270 ng/mL	<500 ng/mL	Normal

**Figure 1:**
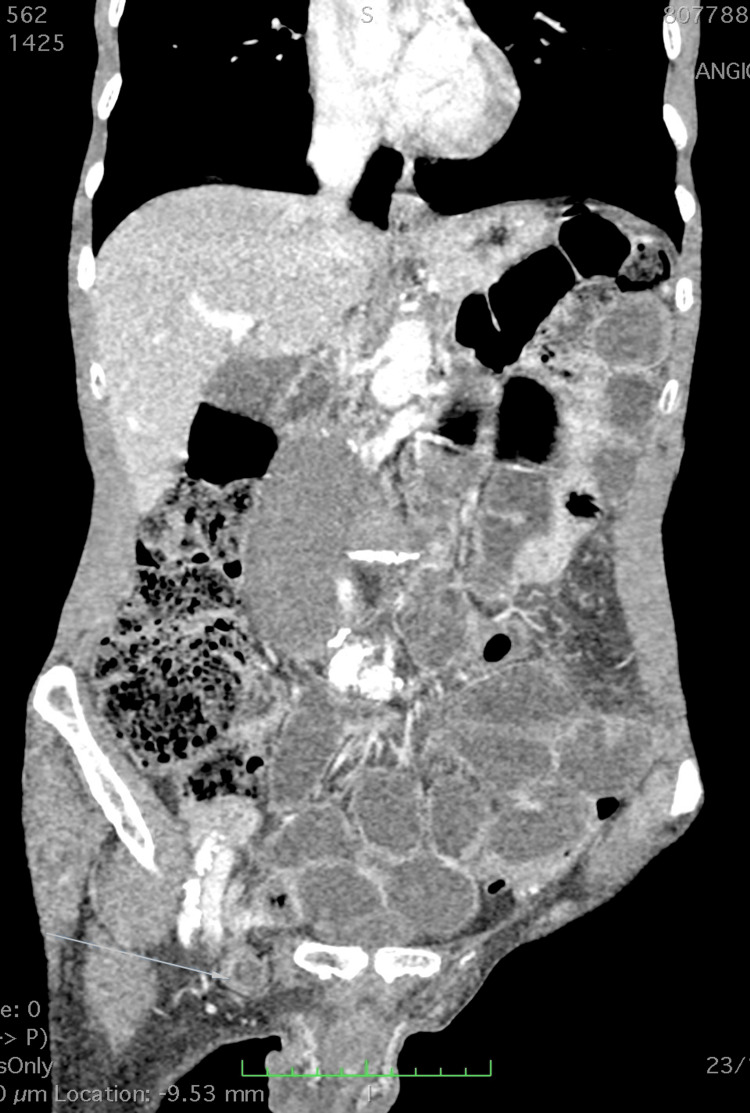
CT scan showing dilated bowel loops and an incarcerated femoral hernia

A computed tomography (CT) scan, as shown in Figure 1, was performed in the emergency department, which revealed dilatation of jejunal-ileal loops extending to the distal ileum, with invagination into the supposedly right inguinal hernia, with wall thickening and mucosal enhancement with contrast and a small amount of free fluid, suggesting intestinal obstruction. It also confirmed the presence of a partially thrombosed fusiform aneurysm of the infrarenal abdominal aorta and noted centrilobular emphysema compatible with COPD.

Intraoperative findings and surgical technique

**Figure 2:**
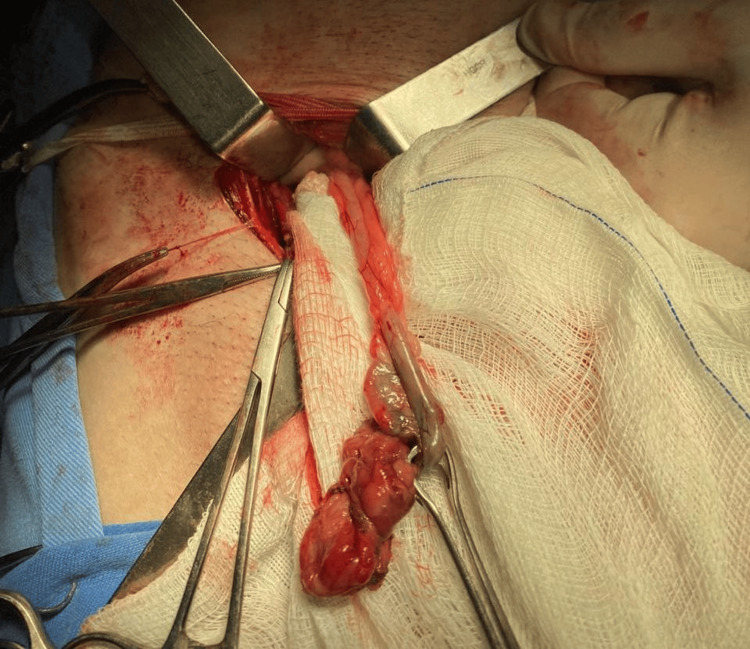
Intraoperative aspect of the appendix with necrosis of the appendiceal tip through the inguinal incision

Given the clinical picture of incarcerated hernia and signs of intestinal obstruction, the patient underwent surgical exploration on 10/23/2025. During the procedure, intraoperative diagnosis revealed a femoral hernia containing the vermiform appendix within the hernia sac, with acute appendicitis and tip necrosis, characterizing a De Garengeot hernia, as shown in Figure 2. The case classification was class 3A [1], indicating acute appendicitis with necrosis of the appendiceal tip.

The surgical technique employed was as follows: Right inguinotomy was performed. Careful dissection by planes until identification of the hernia sac. Opening of the transversalis fascia for better visualization and access to the hernia contents. Appendectomy: The mesoappendix was ligated with 2.0 silk suture, and the appendiceal base was ligated with 2.0 silk, followed by burial of the base with tobacco pouch suture using 3.0 polyglactin. Due to the presence of appendiceal necrosis and associated infectious risk, anatomic repair without prosthetic mesh was chosen. Correction of the femoral hernia was performed using the McVay technique, approximating the conjoint tendon to Cooper's ligament with 2.0 polyglactin sutures. Additionally, a correction similar to the Bassini technique was performed, with approximation of the conjoint tendon to the inguinal ligament with 1.0 polyglactin.

Postoperative course

On postoperative day 1 (10/24/2025), the patient was without complaints, with significant pain improvement and denial of nausea or vomiting. He was discharged from the hospital with a prescription for ciprofloxacin 500 mg every 12 hours and metronidazole 400 mg every eight hours for seven days, plus analgesia as needed.

At outpatient follow-up on 12/01/2025 (40 days postoperatively), the patient showed good evolution, denied pain, bulging, or fever. The surgical scar was in good condition, without bulging on Valsalva maneuvers. He was discharged from the outpatient clinic with guidance for follow-up of the abdominal aortic aneurysm.

Pathological findings

Pathological analysis demonstrated mucosa with an acute inflammatory infiltrate predominantly of neutrophils, extending to the serosa. Foci of hemorrhage, necrosis, and edema in the submucosa were observed. The histopathological conclusion was acute appendicitis.

## Discussion

De Garengeot hernia is a rare surgical condition that represents a diagnostic and therapeutic challenge. Its low incidence, with fewer than 200 cases described, especially when complicated by acute appendicitis, makes each case report valuable to the medical literature. The clinical presentation, as demonstrated in this case, is often nonspecific, with inguinal pain and bulging, mimicking a simple incarcerated hernia. The presence of gastrointestinal symptoms, such as vomiting and constipation, although suggestive of obstruction, is not pathognomonic of appendicitis within the hernia sac.

Preoperative diagnosis of De Garengeot hernia is rare, occurring in less than 10% of cases [1]. CT can be useful, as seen in this patient, in identifying the appendix within the hernia sac and signs of inflammation. However, definitive confirmation is most often intraoperative. The presence of a large infrarenal abdominal aortic aneurysm in this patient added a layer of complexity to management, requiring careful assessment of surgical risk and procedure priority.

The Guenther classification for De Garengeot hernias is crucial for guiding treatment [1]. Our patient fell into class 3A, indicating acute appendicitis with necrosis of the appendiceal tip. This classification is fundamental for the decision to perform an appendectomy and for choosing the type of hernia repair. Appendectomy is universally recommended in cases of acute appendicitis within the hernia sac, regardless of appendiceal status, due to the risk of recurrence and complications [2,3].

A critical aspect of management was the decision to perform anatomic hernia repair without prosthetic mesh. The literature is clear in advising against mesh use in contaminated or potentially contaminated surgical fields, as in acute appendicitis, due to the high risk of mesh infection, fistula, and other serious complications [1,4,5]. The choice of McVay technique combined with principles inspired by the Bassini technique, both anatomic repairs, was appropriate for this scenario, providing robust correction without introducing prosthetic material in an inflamed environment. The excellent postoperative course of the patient, without infectious complications or hernia recurrence on follow-up, corroborates the appropriateness of this approach.

This case highlights the importance of considering De Garengeot hernia in the differential diagnosis of inguinal pain and bulging, especially in elderly patients with comorbidities. High clinical suspicion, combined with complementary imaging studies and meticulous surgical exploration, is essential for successful diagnosis and treatment. Surgical management should be individualized, prioritizing appendectomy and opting for anatomic repairs in cases of acute appendicitis to avoid mesh-related complications.

## Conclusions

De Garengeot hernia with acute appendicitis is a rare condition requiring high clinical suspicion, as preoperative diagnosis is challenging. The present case illustrates the clinical presentation, diagnostic findings, and successful surgical management of a class 3A De Garengeot hernia in an elderly patient with multiple comorbidities. Appendectomy and anatomic hernia repair without mesh are the preferred approaches in scenarios of acute appendicitis within the hernia sac, minimizing the risk of infectious complications and ensuring a favorable outcome. This report reinforces the need for vigilance and an adapted surgical plan for this rare and potentially serious condition.
